# Nicotinic Acetylcholine Receptor α7 Subunit Is an Essential Regulator of Seizure Susceptibility

**DOI:** 10.3389/fneur.2021.656752

**Published:** 2021-04-12

**Authors:** Peng Sun, Da-Gang Liu, Xiang-Ming Ye

**Affiliations:** ^1^Rehabilitation & Sports Medicine Research Institute of Zhejiang Province, Zhejiang Provincial People's Hospital, People's Hospital of Hangzhou Medical College, Hangzhou, China; ^2^Department of Neurosurgery Medicine, Weihai Central Hospital, Weihai, China

**Keywords:** cholinergic receptors, α7 nicotinic acetylcholine receptors, epilepsy, seizure susceptibility, CA1 pyramidal neuron

## Abstract

A large body of data has confirmed that α7 nicotinic acetylcholine receptors (nAChRs) play a pivotal role in cognition, memory, and other neuropsychiatric diseases, but their effect on seizure susceptibility in C57BL/6 wild-type mice is not fully understood. Here, we showed that decreased activity of α7 nAChRs could increase the excitability of CA1 pyramidal neurons and shorten the onset time of epilepsy in pilocarpine-induced mouse models. However, compared with the control group, there was no apparent effect of increasing the activity of α7 nAChRs. Moreover, the expression of α7 nAChRs is downregulated in human epileptogenic tissues. Taken together, our findings indicate that α7 nAChR is an essential regulator of seizure susceptibility.

## Introduction

Nicotinic acetylcholine receptors (nAChRs) are a family of ligand-gated, pentameric ion channels. Understanding the precise roles of nAChRs remains a challenge because they can modulate cholinergic activities both postsynaptically and presynaptically (1). In humans, there are 16 different subunits (α1-7, α9-10, β1-4, δ, ε, γ) of nAChRs, which can be found both in the peripheral and central nervous systems (2–4). Of these, 12 are associated with a wide spectrum of physiological and pharmacological functions (5). The main function of nAChRs is to regulate neuronal plasticity (6) and neuroprotection (7, 8), and the most abundant nAChR subunits are the α4β2 and α7 subunits within the central nervous system. Mutations in the transmembrane regions of the neuronal α4β2 subunit receptors in the neocortex and thalamus can cause autosomal dominant nocturnal frontal lobe epilepsy (9), which is a focal epilepsy in the frontal lobe with attacks typically arising during non-rapid eye movement sleep (10). The α7 subunit is expressed widely in the brain with its highest levels observed in the hippocampus and cortex (11), and its role has been associated with both impairment in cognition and neuropsychiatric phenotypes (12–15).

Our previous studies have indicated that activation of α7 nAChR could decrease seizure susceptibility in Chat-Mecp2^−/*y*^ mice (16), but the role of α7 nAChR in seizure susceptibility in C57BL/6 wild-type mice and humans remains uncertain. In the present study, we explored the function of α7 nAChRs in seizure susceptibility in wild-type mice. We revealed that decreased α7 nAChR activity in CA1 parvalbumin (PV) neurons in the hippocampus could increase seizure susceptibility in wild-type mice, while the increased activity of α7 nAChR had no significant effect on seizure susceptibility. We also tested the expression of α7 nAChR that was downregulated in tissues of humans, experiencing epilepsy after traumatic brain injury or intracerebral hemorrhage. Our findings suggest that α7 nAChR is an essential regulator of seizure susceptibility.

## Materials and Methods

All human material was reviewed and approved by the Medical Ethical Committee of Zhejiang Provincial People's Hospital, People's Hospital of Hangzhou Medical College (No. 2019KY255) based on the Code of Ethics of the World Medical Association. All participants provided written informed consent. All animal experimental procedures were examined and approved by the Animal Advisory Committee of Zhejiang Provincial People's Hospital, the People's Hospital of Hangzhou Medical College based on the National Institutes of Health Guide for the Care and Use of Laboratory Animals.

### Animals and Reagents

We used C57BL/6 wild-type mice to complete the experiment. For acute pilocarpine-induced epilepsy models, C57BL/6 wild-type male mice weighing 25–30 g were used. Before the behavioral test, we kept the mice in a 12/12 h light/dark cycle, and the environmental conditions were controlled. Mice with abnormal body weight and appearance were excluded from the behavioral test. Sexually dimorphic observations were not observed. The primary antibodies used were rabbit polyclonal anti-α7 nAChR (Santa Cruz Biotechnology, USA, Cat#: sc-5544), and rabbit polyclonal anti- glyceraldehyde 3-phosphate dehydrogenase (GAPDH) (Cell Signaling Technology, Cat#:5014S). MLA was purchased from Abcam (UK), while pilocarpine and PNU282987 were purchased from Sigma-Aldrich (USA).

### Immunohistochemistry

We anesthetized the mice and perfused their brain vessel with 4% paraformaldehyde dissolved in phosphate-buffered saline (PBS). We cut and fixed the whole brains in paraformaldehyde at 4°C overnight and further dehydrated the brains in 30% sucrose in PBS. According to anatomical landmarks, brain sections were cut 35-μm-thick from the regions of interest using a freezing microtome. We treated the brain sections with 10% (v/v) normal donkey serum for immunolabeling in PBS containing 0.3% Triton X-100. Subsequently, we incubated the brain sections with antibodies against anti-α7 nAChR (1:100) at 4°C overnight. We visualized the immunoreactivity with the secondary antibodies of Alexa Fluor 594 donkey anti-mouse, Alexa Fluor 633 donkey anti-goat, and Alexa Fluor 488 donkey anti-rabbit IgG (1:400). We visualized the immunofluorescent images by confocal microscopy (FV1000 Laser scanning confocal microscope, USA).

### Human Tissue Preparation and Western Blot Analysis

We dissected 1 g of epilepsy tissue from fresh frozen brain sections that were maintained at −80°C. The tissue was homogenized in a lysis buffer (Beyotime Biotechnology, China) containing 1 mM protease inhibitor phenylmethylsulfonyl fluoride (PMSF) (Beyotime Biotechnology). We collected the supernatants after centrifugation for 10 min at 15 000 rpm. After the protein concentration was measured using Bradford's solution (Beyotime Biotechnology), we boiled the samples in a loading buffer containing equivalent amounts of protein for 5 min (Beyotime Biotechnology). We performed sodium dodecyl sulfate-polyacrylamide gel electrophoresis and transferred proteins to an Immobilon polyvinylidene fluoride (PVDF) membrane (Millipore) for 100 min at 300 mA. We incubated the membranes with primary antibodies against anti-α7 nAChR (1:100) after blocking with Tris-buffered saline buffer with Tween 20 (TBST) solution (50 mM Tris-HCl, pH 7.5, 150 mM NaCl, and 0.1% Tween 20) containing 5% skimmed milk for 1 h at room temperature. After washing with TBST, we incubated the blots with secondary antibodies (1:7,500) for 1 h at room temperature. We detected the signals with enhanced chemiluminescence and developed them on an X-ray film. We digitized the immunoblots on a flatbed scanner and quantified the images using the US National Institutes of Health Image program for densitometric quantification.

### Surgeries and Electroencephalogram Measurements

We secured the mice in a stereotactic head frame and made an incision along the midline after the mice were anesthetized with isoflurane. We used 8% H_2_O_2_ to observe the bregma and the posterior. We placed the electrode over the cranium with three screws (three positions: the first screw (left): A-P: +1.5 mm, lateral: +1.5 mm; the second and the third screws: A-P: −3 mm, lateral: ±3 mm). We placed the cannula (intraventricular drug administration) into the CA1 in the hippocampus (position (right): A-P: −1.94 mm, lateral: ±1.1 mm depth: −2.0 mm). We recorded the electroencephalogram (EEG) results in freely moving mice for 1 week after the surgery. α7 nAChR agonist (PNU282987, a selective α7 nAChR agonist, 1M) and antagonist (methyllycaconitine citrate (MLA), a specific α7 nAChR blocker,100 nM) were injected into the hippocampus in the CA1 by microtubule drug administration. Pilocarpine was injected into the peritoneal cavity following intraperitoneal administration.

We defined the abnormal wave as an amplitude >400 mV. We assessed the pilocarpine-induced seizure stages as follows: onset time was measured from the moment the C57BL/6 mice were injected with pilocarpine until the first epileptic waves were observed. The latency of generalized tonic-clonic (GTC) seizure was measured from the moment the C57BL/6 mice, were injected with pilocarpine until the first GTC seizure epileptic waves were observed. The latency of death was measured from the moment the mice were injected with injection pilocarpine, until the death of the mice.

### Electrophysiology

Coronal slices of the hippocampus were performed in approximately 4- weeks-old mice. Slices (300 μm) were prepared with a Vibroslice (Leica VT 1000S) in ice-cold artificial cerebrospinal fluid (ACSF: 125 mM NaCl, 3 mM KCl, 1.25 mM NaH2PO4, 2 mM MgSO4, 2 mM CaCl2, 25 mM NaHCO3 and 10 mM glucose). After recovery for ~60 min, incubation in ACSF at 33°C was followed by ~60 min at 22°C, when slices were transferred to the recording chamber and superfused (3 mL min^−^) with ACSF at 32–33°C. All solutions were saturated with 95% O_2_ and 5% CO_2_. The neurons were identified in the brain sections using an upright microscope equipped with a 40× water-immersion lens (Nikon, Eclipse FN1, Japan), and the electrical activity was recorded using whole-cell technology (MultiClamp 700-B Amplifier, Digidata 1440 A analog-to-digital converter, and **PCLAMP** 10.2 software, Axon Instruments Molecular Devices, USA). Pyramidal neurons were recorded in the hippocampal CA1 region. Glass pipettes (3–4.5 MΩ) used for whole-cell recording were filled with internal recording solution: 110 mM K-gluconate, 40 mM KCl, 10 mM HEPES, 2 mM Mg2ATP, 0.5 mM NaGTP, and 0.2 mM EGTA; pH was adjusted to 7.25 with 10 M KOH. We also added DL-2-amino-5-phosphonovaleric acid (DL-AP5; 50 μM, Tocris Bioscience), 6, 7-dinitroquinoxaline-2, 3-dione (DNQX; 20 μM, Tocris Bioscience) and picrotoxin (100 μM, Abcam), to block AMPA-mediated, NMDA-mediated, and GABA-mediated synaptic transmission.

### Statistical Analysis

Unless otherwise stated, we expressed the data as mean ± standard error of the mean (SEM); error bars show the SEM (*n* = number of samples). A two-tailed Student's *t*-test was used to compare the means from the same group of cells. The differences between more than two groups were tested with two-way analysis of variance. Differences were considered significant at *P* < 0.05.

## Results

### Expression of α7 nAChR in the CA1 Neurons in the Hippocampus

**Figure 1:**
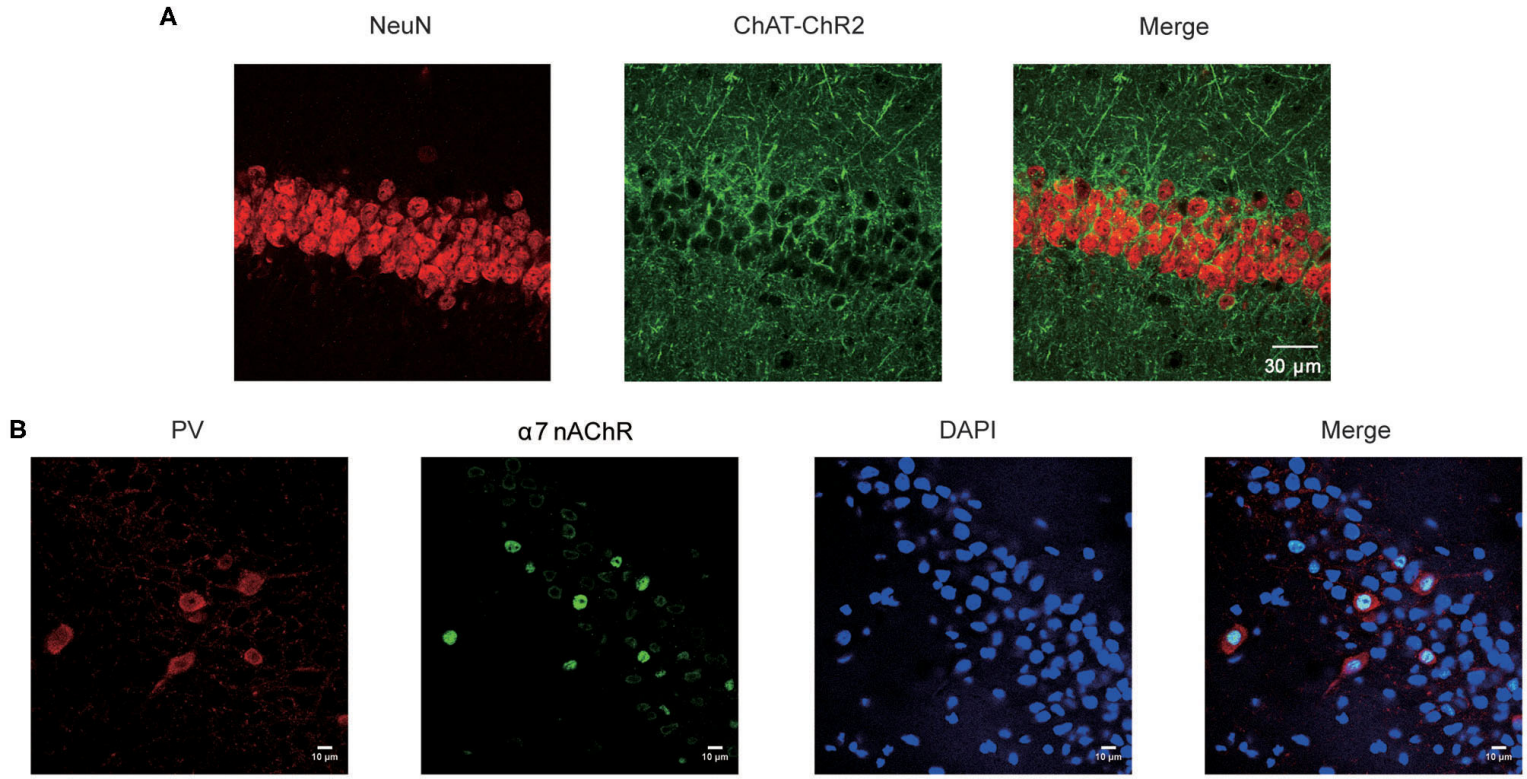
Expression of α7 nAChR in the CA1 neurons in hippocampus. **(A)** Fluorescence images showing CA1 sections stained for the NeuN and the cholinergic neuron projections in the Chat-ChR2-EYFP mice. **(B)** Immunostaining of the hippocampal pyramidal layer with neurons and α7 nAChRs, showing the expression pattern of α7 nAChRs on PV GABAergic neurons and non-PV neurons.

Mice expressing the channelrhodopsin-2 (ChR2) protein under the control of the choline acetyltransferase (ChAT, a marker for cholinergic neurons) promoter (ChAT–ChR2–EYFP) were used in this study. The hippocampus has been reported to be the main target for the projection of basal forebrain cholinergic neurons (17). To confirm this, we stained the NeuN in hippocampal CA1 neurons in ChAT–ChR2–EYFP mice (Figure 1A. left). We found that there are lots of cholinergic neuron fiber projections in this area (Figure 1A. middle).

After merging, the results showed that hippocampus CA1 neurons receive dense cholinergic projections (Figure 1A. right). Previous studies showed that the main target projection neurons of α7 nAChRs are the PV-positive interneurons, which are the most prominent GABAergic neurons in the hippocampus (16). Therefore, we stained α7 nAChRs in the CA1 neurons (Figure 1B). The results showed that α7 nAChRs were concentrated in PV GABAergic neurons, with little expression in non-PV neurons in the CA1 (Figure 1B). Taken together, these results indicate that the cholinergic neurons were highly expressed in the hippocampal CA1 PV GABAergic neurons.

### Decreased Activity of α7 nAChRs Could Increase the Seizure Susceptibility in C57Bl/6 Wild-Type Mice

To investigate the effects of α7 nAChRs on seizure susceptibility in C57BL/6 wild-type mice, we injected PNU282987 (1 M) and MLA (100 nM) into the hippocampus in the CA1 by microtubule drug administration. Scopolamine methylnitrate (1 mg/kg s.c.; Sigma) was administered 30 min before pilocarpine injection to avoid peripheral cholinergic effects (18). We measured seizure susceptibility by injecting the cholinergic agonist pilocarpine (250 mg/kg i.p.) following intraperitoneal administration, which acts on muscarinic receptors.

**Figure 2:**
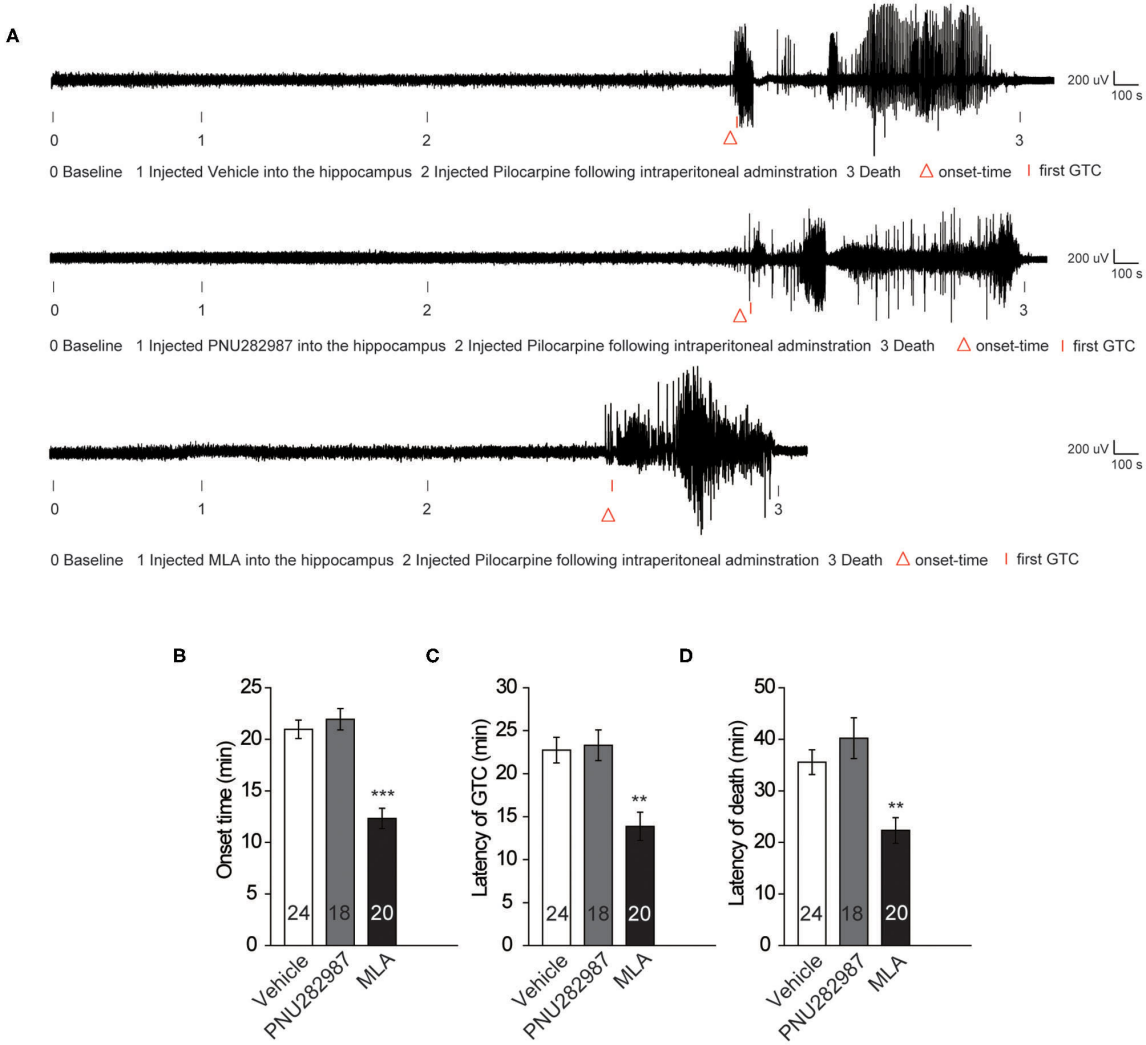
Decreased activity of α7 nAChRs could increase the seizure susceptibility in C57BL/6 wild-type mice. **(A)** Representative compressed electroencephalogram from a cortical lead depicting an electrographic seizure in a C57BL/6 mouse with seizure behaviors identified at the time of occurrence. Background EEG baseline is shown before seizure onset. **(B–D)** Summary histograms of the onset time, latency of GTC, and latency of death, respectively. Two-way ANOVA was used. ***P < 0.001; **P < 0.01; vs. to vehicle. Error bars are means ± s.e.m.

To observe the electrographic changes caused by pilocarpine-induced seizures between the vehicle and α7 nAChR agonist and antagonist, we recorded EEGs using bilateral epidural screw electrodes (see **Methods** section) and quantified the electrographic seizures by the latency of onset time, the latency of GTC, and the latency of death. After 10 min baseline recording, we administrated the vehicle and PNU282987 in the CA1 of the hippocampus in C57BL/6 wildtype mice. After recording the EEGs for 15 min, we injected pilocarpine following intraperitoneal administration to induce epilepsy. The results showed that there was no significant difference in the onset time of epilepsy and the latency of GTC (Figures 2A–C). Moreover, we found the latency of death was slightly prolonged after administration of PNU282987, but there was no significant difference between the vehicle-treated control group and the C57BL/6 wild-type group (Figure 2D). However, the latency of onset time, the latency of GTC, and the latency of death were significantly shortened with MLA treatment (Figures 2A–D). The results indicated that a decrease in the activity of α7 nAChRs could increase seizure susceptibility but the increased activity of α7 nAChRs has no effect.

### Decreased Activity of α7 nAChRs Could Increase the Excitability of the CA1 Pyramidal Neurons in the Hippocampus in C57BL/6 Wild-Type Mice

**Figure 3:**
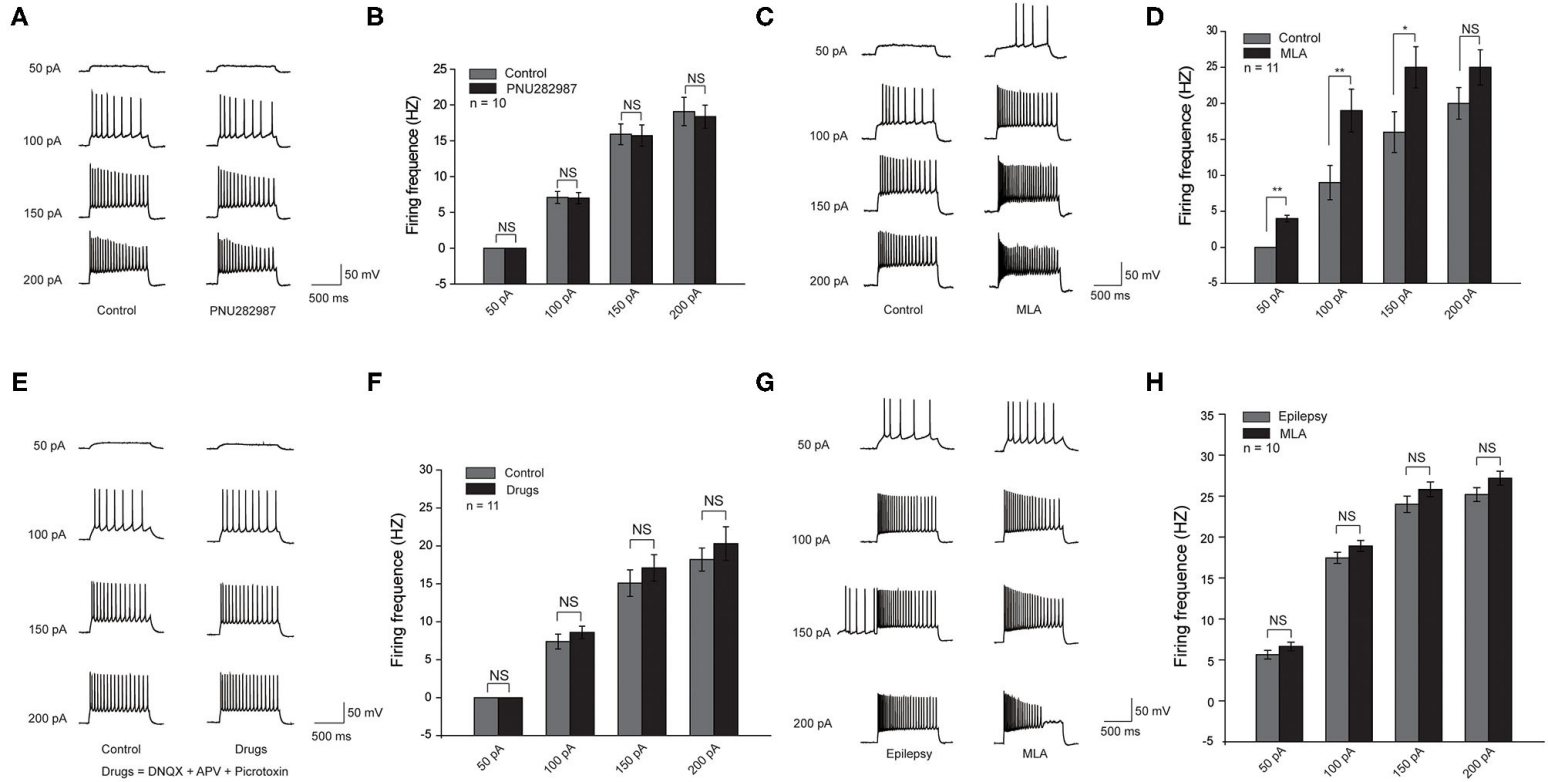
Decreased activity of α7 nAChRs could increase the excitability of the CA1 pyramidal neurons in the hippocampus in C57BL/6 wild-type mice. **(A,C,E,G)** Voltage responses of a representative to in pyramidal neurons to various injection steps (1s) from top to bottom, 50, 100, 150, 200 pA. **(A)** Left: Control group. Right: PNU282987 group. **(B)** Summary histogram showing the number of pyramidal neuron APs. Two-way ANOVA was used. *n* = 10. **(C)** Left: Control group. Right: MLA group. **(D)** Summary histogram showing the number of pyramidal neuron APs. Two-way ANOVA was used. *n* = 11. **(E)** Left: Control group. Right: Drugs group (Drugs = DNQX + APV + Picrotoxin). **(F)** Summary histogram showing the number of pyramidal neuron APs. Two-way ANOVA was used. *n* = 11. **(G)** Left: Epilepsy group. Right: MLA group. **(H)** Summary histogram showing the number of pyramidal neuron APs. All pyramidal neuron membrane potential was kept at −70 mV by injecting a small DC current through the recording pipette. *P*-value was calculated by two-sided *t*-test. **P < 0.01; *P < 0.05. Error bars are means ± s.e.m.

To further demonstrate the effect of α7 nAChRs, we recorded the excitability of CA1 pyramidal neurons by checking the number of action potentials (APs) elicited by current injections of various amplitudes (1 s, 0 to +250 pA) in the acute cortical slices. All pyramidal neurons showed high-frequency discharges with increasing currents. We assessed the effect of PNU282987(1 μm), a selective α7 nAChR agonist, on hippocampal slices from C57BL/6 mice. In contrast to the control group, there was no significant change in the excitability of pyramidal neurons in C57BL/6 wide-type mice (Figures 3A,B). However, with the bath application of MLA (10 nM), a specific α7 nAChR blocker, the number of APs was significantly higher in the C57BL/6 group compared with that in the control group (Figures 3C,D). Taken together, these results revealed that a decrease in the activity of α7 nAChRs could increase the excitability of CA1 pyramidal neurons in the hippocampus.

We believed that the effect of α7 nAChRs on pyramidal neuron excitability could be due to the following two possibilities: the inhibitory input from parvalbumin (PV) interneurons to pyramidal neurons, and the direct modulation of α7 nAChRs on pyramidal neurons (19). To distinguish between the two possibilities, we administered DL-APV, DNQX, and picrotoxin to block NMDA-mediated, AMPA-mediated, and GABAA-mediated synaptic transmission, respectively. The results showed that there was a slight increase but no significant change of pyramidal neuron excitability after the application of MLA (10 nM), suggesting that the excitability of pyramidal neurons is mostly regulated by the inhibitory input from PV neurons (Figures 3E,F).

To further study the effect of MLA on the excitability of CA1 pyramidal neurons in epileptic brain slices. We recorded the excitability of CA1 pyramidal neurons in epileptic brain slices before and after administrating MLA. The excitability of pyramidal neurons was increased after epilepsy, and the excitability is slightly higher after adding MLA. However, there was no significant difference in pyramidal neuron excitability after the application of MLA (Figures 3G,H). The results indicate that the α7 nAChRs on pyramidal neurons may also play a role in regulating excitability.

### Decreased α7 nAChR Expression in Human Epileptogenic Tissues

**Figure 4:**
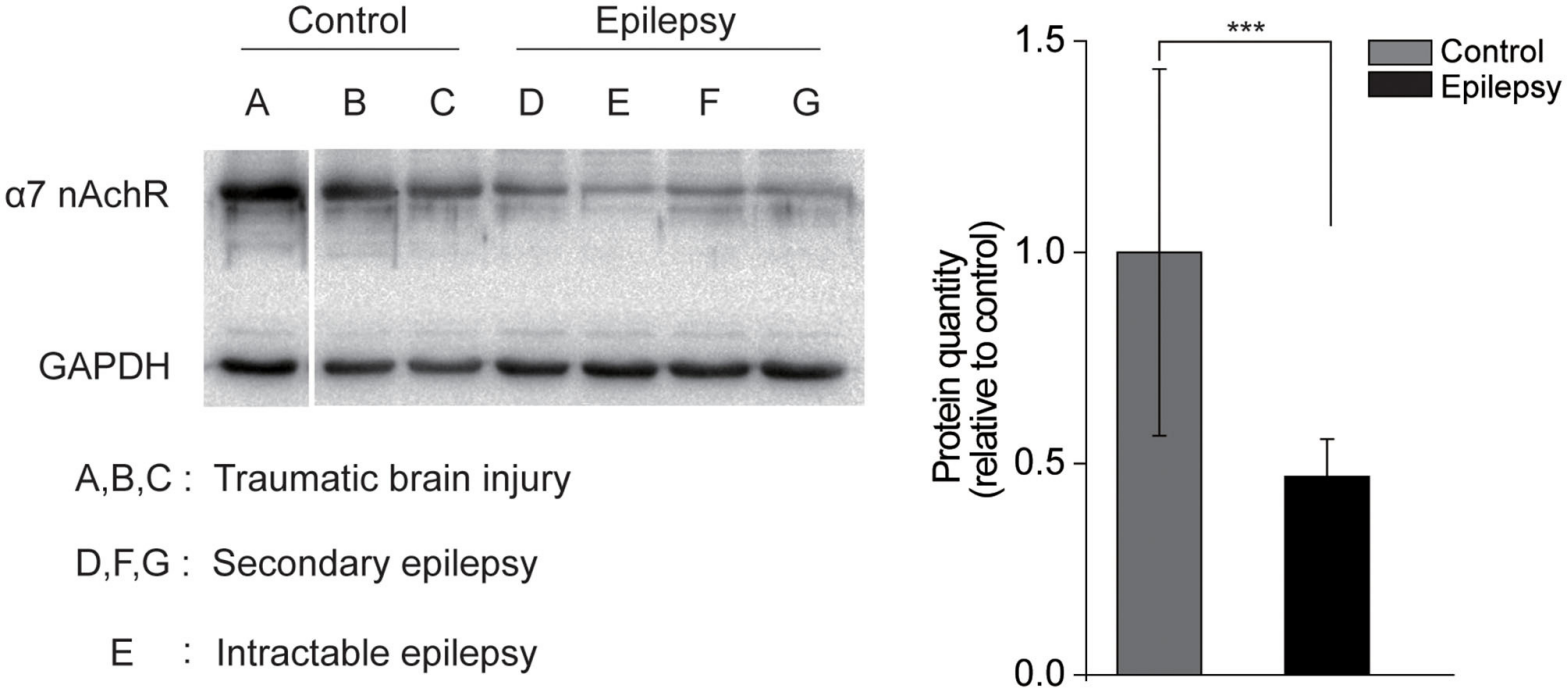
Decreased α7 nAChRs expression in human epileptogenic tissue. **(A)** Immunoblotting with α7 nAChRs in the membrane fraction of cortex from control individuals or individuals with epilepsy. Each lane was loaded with 40 μg of protein with GAPDH as the loading control. **(B)** Quantification of α7 nAChRs proteins in control and epilepsy groups. *n* = 3. Protein levels of epilepsy tissue were normalized to the corresponding ones of non-epilepsy tissue. ***P < 0.001; Error bars are means ± s.e.m.

To further confirm the association between the activity of α7 nAChRs and seizure susceptibility, we assessed the expression of α7 nAChRs in cell membranes by immunoblotting from the epileptogenic foci tissue between subjects with secondary and intractable epilepsy and the those with traumatic brain injury (Figure 4A). The results showed that the level of α7 nAChRs in the epilepsy samples was only ~50% of that in the control group (Figure 4B). This observation is also supported by a decrease in α7 nAChR expression in human epileptogenic tissues.

## Discussion

This study investigated the association between the activity of α7 nAChRs and seizure susceptibility. We presented evidence that decreased activity of α7 nAChRs could increase seizure susceptibility in C57BL/6 wild-type mice. First, we proved that cholinergic neurons exist, and its subunit receptor-α7 nAChRs mostly expressed in CA1 PV interneurons. Second, we studied seizure susceptibility after the stimulation and inhibition of α7 nAChRs. To address this issue, we used C57BL/6 wild-type mice experiencing pilocarpine-induced seizures as the experimental animals. The results showed that the seizure susceptibility between the PNU282987-treated mice and the control group was not significantly different, but the MLA-treated mice could significantly shorten the seizure susceptibility. Third, the number of APs was significantly increased after MLA treatment; however, there were no significant differences after infusion of PNU282987 in CA1 pyramidal neurons. Furthermore, we found that the excitability of pyramidal neurons was mostly regulated by α7 nAChRs receptors of PV GABAergic neurons. Finally, the study showed that the expression of α7 nAChR protein was reduced in the epileptogenic foci tissue from individuals. In summary, our study identified that decreased α7 nAChRs may be a risk factor for seizure susceptibility.

It has been reported that α7 nAChRs are widely expressed in the hippocampus and thalamus, but they are observed at low levels in the postmortem human brain (20, 21). The α7 nAChRs are concentrated on almost all synapses both presynaptically and postsynaptically in the CA1 region of the hippocampus (22). The initial evidence of α7 nAChR activity in synapses was obtained from rat hippocampal CA1 pyramidal neurons by examining rats' spontaneous activity (23–25). Loss of α7 nAChRs may play a pivotal role in cognitive function damage (26–29). Previous studies have shown that α7 nAChRs have neuroprotective effects (30). The administration of nicotine may attenuate microglial activity and increase the eclampsia-like seizure threshold in the rat hippocampus through the α7 nicotinic receptor (31). This revealed the influence of α7 nAChRs on the threshold in pregnant rats. Previous studies have shown that activation of α7 nAChRs could decrease the seizure susceptibility in Chat-Mecp2^−/*y*^ mice (16). Therefore, we hypothesized that α7 nAChRs may be associate with epilepsy. In this present study, immunofluorescence results on hippocampal neurons showed that α7 nAChRs are expressed in pyramidal and PV neurons in the hippocampus. Previous studies showed that PV interneuron axons project to the peribody area of pyramidal neurons and regulate the activity of pyramidal neurons (32). We revealed that treatment with MLA to block α7 nAChRs on CA1 PV interneurons indirectly increased the excitability of these neurons. Further studies are required to examine whether α7 nAChRs in pyramidal neurons affect its excitability. This study only tested the role of α7 nAChRs in the pilocarpine model, and further studies are required to confirm its effects in more epilepsy models.

Decreased excitability of α7 nAChRs on CA1 pyramidal neurons in the hippocampus leads to high seizure sensitivity in mice. α7 nAChR antagonists have been reported to alter the frequency and amplitude of glutamatergic neurons recorded from pyramidal neurons in hippocampal samples obtained from patients with mesial temporal lobe epilepsy with hippocampal sclerosis (33). The results of this study also suggested that the application of α7 receptor antagonists in patients with temporal lobe epilepsy could overexcite pyramidal neurons, a result similar to that of our study. A recent study also demonstrated that the α7 nAChR agonist choline chloride could improve epilepsy, depression, and memory deficits in the PTZ-kindled mouse model (34). This also suggests that α7 nAChRs play a pivotal role in epilepsy. Moreover, in our study, the expression of α7 nAChRs was reduced in human epileptogenic tissues. Our findings suggest that the downregulation of α7 nAChRs contributes to human epilepsy. However, the increased activity of α7 nAChRs on CA1 pyramidal neurons in the hippocampus had no significant effect on seizure sensitivity. Earlier studies also showed that selective allosteric modulators of α7 nAChRs could have potential therapeutic applications in epilepsy (35). Overall, our results suggest that α7 nAChRs are an essential regulator of seizure susceptibility.

## Data Availability Statement

The datasets generated for this article are not readily available because this would jeopardize patient privacy. Requests to access the datasets should be directed to sp120@zju.edu.cn.

## Ethics Statement

The studies involving human participants were reviewed and approved by Medical Ethical Committee of Zhejiang Provincial People's Hospital, People's Hospital of Hangzhou Medical College. The patients/participants provided their written informed consent to participate in this study. The animal study was reviewed and approved by Medical Ethical Committee of Zhejiang Provincial People's Hospital, People's Hospital of Hangzhou Medical College. Written informed consent was obtained from the owners for the participation of their animals in this study.

## Author Contributions

PS conducted the immunohistochemistry, western blot analysis, electrophysiology experiments, analyzed data, and wrote the manuscript. D-GL conducted the animal behavioral studies, EEG recordings, and collected the data. X-MY supervised all phases of the project and approved the final manuscript. All authors contributed to the article and approved the submitted version.

## Conflict of Interest

The authors declare that the research was conducted in the absence of any commercial or financial relationships that could be construed as a potential conflict of interest.
